# Lactate-induced IGF1R protein lactylation promotes proliferation and metabolic reprogramming of lung cancer cells

**DOI:** 10.1515/biol-2022-0874

**Published:** 2024-05-29

**Authors:** Rong Zhang, Lulu Li, Junyan Yu

**Affiliations:** Department of Oncology, Heping Hospital Affiliated to Changzhi Medical College, No.110, Yan’an South Road, Luzhou District, Changzhi City, Shanxi Province, 046000, China

**Keywords:** lung cancer, IGF1R, IGF1, lactylation, glycolysis

## Abstract

Lung cancer (LC) is regarded as a fatal cancer, and insulin-like growth factor 1 (IGF1) and its receptor (IGF1R) have been found to play a key role in regulating tumor glycolytic metabolism. The aim of this study is to investigate LC proliferation regulated by metabolite-mediated IGF1R lactylation. IGF1R was highly expressed in LC tissues and cells, and the effects of IGF1R on protein stability were inhibited by Lactate dehydrogenase A (LDHA) inhibition. Moreover, the tightness of IGF1R binding to IGF1 was also enhanced by exogenous lactic acid but suppressed by LDHA silencing, while cell viability and proliferation were promoted by over-expression of IGF1R. Exogenous lactic acid further exacerbated the effects of the IGF1R gene, while LDHA knocking down reduced the IGF1R-induced malignant behaviors. The IGF1R and exogenous lactic acid were also found to increase extracellular acidification rate (ECAR) and decrease oxygen consumption rate to regulate glycolysis, which was inhibited by LDHA deficiency in LC cells. The study concluded that IGF1R-mediated aggressive behaviors of LC cells were associated with higher levels of IGF1R lactylation. Moreover, lactic acid can improve the protein stability of the IGF1R oncogene, thus promoting glycolysis and generating lactic acid, forming a closed loop. Therefore, targeting IGF1R is envisaged to provide a novel strategy for developing therapeutic agents against LC.

## 1 Introduction

Lung cancer (LC) is the primary cause of death associated with tumors globally, posing a significant hazard to human health. Mounting evidence indicates that glycolytic metabolism plays a significant role in cancers, including LC, and growth and metastasis of LC inhibition can be achieved by targeting the gene associated with glycolysis [1,2].

Insulin-like growth factor 1 (IGF1) and its receptor (IGF1R) have been reported to play a key role in regulating tumor glycolytic metabolism, where IGF1 has been found to stimulate IGF1R to increase tumor growth and glycolysis in pancreatic cancer [3]. IGF1 plays an additional carcinogenic role in pancreatic ductal adenocarcinoma by reducing the acetylation modification level of ENO2, a key glycolytic enzyme [4], while IGF1R expression inhibition has been found to suppress glycolysis in hepatocellular carcinoma [5]. Moreover, IGF1R has also been reported to inhibit glycolysis in LC [6,7]. Hence, targeting IGF1R serves as a promising strategy for treating LC.

Glucose-generated lactic acid plays an important role in tumors as an energy source in the process of glycolysis, promoting angiogenesis, promoting tumor cell invasion and metastasis, and facilitating tumor cell immune escape. Research has demonstrated that lactic acid can facilitate LC progression, participate in regulating glycolysis and mitochondrial metabolism of LC cells [8], and induce the release of Mg^2+^ in the endoplasmic reticulum to participate in tissue damage [9]. Lactate dehydrogenase A (LDHA) is a significant enzyme in the glycolysis pathway that catalyzes the conversion of pyruvate into lactic acid. Previous research has demonstrated that LDHA levels are elevated in different types of tumors, and it has a significant role in regulating glycolysis, where downregulating the expression of LDHA had been found to significantly inhibit the proliferation and promote the apoptosis of tumor cells [10–12].

Therefore, this study attempted to investigate IGF1R implications in LC and whether the IGF1R modulation is related to glycolysis.

## 2 Materials and methods

### 2.1 Tissue collection

Forty-five pairs of LC tissues and the matched adjacent tissues were obtained from patients with LC diagnosed in Heping Hospital Affiliated to Changzhi Medical College. All enrolled subjects had not received chemotherapy or radiation before surgery. The tissues were stored immediately at −80 °C for subsequent use.


**Informed consent:** Informed consent has been obtained from all individuals included in this study.
**Ethical approval:** The research related to human use has been complied with all the relevant national regulations, institutional policies and in accordance with the tenets of the Helsinki Declaration, and has been approved by the Ethics Committee of Heping Hospital Affiliated to Changzhi Medical College.

### 2.2 Bioinformatic analysis

The mRNA expression of IGF1R in samples of 501 lung squamous cell carcinoma (LUSC) tissues and 49 normal tissues were obtained from the Encyclopedia of RNA Interactomes (ENCORI) online platform (http://starbase.sysu.edu.cn/index.php). The protein expression of IGF1R in samples of 110 LUSC tissues and 102 normal tissues were obtained from the University of Alabama at Birmingham CANcer data analysis Portal (UALCAN) database (https://ualcan.path.uab.edu).

### 2.3 Cell lines and cell culture

The human lung bronchial epithelial cell 16HBE and LC cell lines including PC9, A549, H460, and H1299 used in this study were purchased from the Cell Bank, Chinese Academy of Sciences. All cells were cultured in RPMI 1640 medium containing 10% fetal bovine serum (Gibco, 23400-021) and placed in an incubator at 37℃, 5% CO_2_ and 95% humidity. The PC9 and A549 cells (1 × 10^4^ cells/well) were inoculated in six-well plates and treated with lactic acid (15 mM, Sigma) for 24 h [13]. HEK293T cells were cultured in DMEM supplemented with 10% FBS, 2 mM glutamine, and 1% penicillin and streptomycin.

### 2.4 Cell transfection

In order to downregulate LDHA in PC9 and A549 cells, the small interfering RNA of LDHA (si-LDHA) and the negative control (si-NC), the adenovirus plasmid pAdTrack-CMV of IGF1R cDNA (IGF1R), and the empty control (vector) obtained from GenePharma were transfected into cells. In brief, cells with the density of 5 × 10^4^ cells/well were seeded in the 24-well plates. The transfection was performed with the help of Lipofectamine 3000 (Invitrogen). The siRNA sequences are: si-LDHA: GGCAAAGACUAUAAUGUAACU (sense), UUACAUUAUAGUCUUUGCCAU (antisense); si-NC: UUCUCCGAACGUGUCACGUTT (sense), ACGUGACACGUUCGGAGAATT (antisense).

### 2.5 Reverse-transcription quantitative real-time polymerase chain reaction (RT-qPCR)

RNA was extracted from tissues and cells using TRIzol kit (Invitrogen), and was reverse-transcribed into cDNA according to PrimeScript RT kit instruction (Takara). IGF1R expression was detected by qRCP. The reaction conditions were as follows (40 cycles): 95℃ for 30 s, 95℃ for 15 s, and 60℃ for 30 s. The melting curve and amplification curve were obtained after the reaction. β-actin was used as the internal reference for the relative expression of IGF1R (sense: GTGGGGGCTCGTGTTTCTC, antisense: GATCACCGTGCAGTTTTCCA) using an optimized comparative Ct (2^ΔΔCt^) value method.

### 2.6 Western blot

Tumor tissues and adjacent tumor tissues were added with 200 μL of lysate for 30 min, then the mixture was centrifuged (12,000 rpm, 4℃), and the supernatant was collected for use. For cell lines, all operations are performed on ice, and the procedures are the same as for tissue extraction. The total protein content was determined using a BCA quantitative kit, and the centrifuged supernatant was turned on SDS-PAGE for 2 h. Then, 10 μL of samples were taken for electrophoresis at 80–120 V and transferred to PVDF membranes (Millipore). The membranes were blocked with 5% skim milk for 2 h. Membranes were then incubated with primary antibodies at 4°C overnight, followed by incubation with secondary antibody for 1 h. Band signals were visualized using an ECL kit, and exposed in a ChemiDocTM XRSC System (Bio-Rad). The information of all antibodies are listed as follows: anti-IGF1R antibody (ab182408, 1/1,000) and anti-IGF1 antibody (ab9572, 0.1 µg/mL) were purchased from Abcam. Anti-l-Lactyl Lysine Rabbit pAb (PTM-1401, 1/500) was obtained from PTM Biolabs. Moreover, cycloheximide (CHX, 150 µg/mL) treated on PC9 and A549 cells for 0, 6, 12, and 24 h was used for inhibition of protein synthesis [14].

### 2.7 Immunoprecipitation (IP) and Co-IP

The IP method was conducted for purification of IGF1R protein from tissues and cell lines. The specific process is as follows: the anti-IGF1R antibody was mixed with the extracted protein (400 μg) at 4℃ overnight. The incubated sample was mixed with protein A/G magnetic beads (Bimake) and incubated for 1 h. After washing the beads with PBST for 3 times, the captured proteins were eluted with SDS loading buffer. Western blot was performed with anti-l-Lactyl Lysine Rabbit pAb to detect the lactic modified IGF1R level.

Co-IP assays were performed to detect the interaction between IGF1R and IGF1 using protein A/G magnetic beads. The pre-cooled IP cell lysate of HEK293T cells containing protease inhibitors was added into the collected cells (4℃, 30 min), centrifuged, and determined by BCA method. The supernatant was denatured and used for input. IgG (1.0 μg) and protein A/G beads (20 μL) were added to the IgG group, and 20 μL protein A/G beads were added to the IP group, and incubated at 4℃ for 1 h. After centrifugation, antibody was added to the supernatant at 4℃ and incubated overnight. The IP complex was collected after centrifugation, and washed with pre-cooled IP lysate (without inhibitor) for four times. After each centrifugation, the supernatant was collected, and 80 μL loading buffer was added. Finally, the supernatant (10 μL) which has been boiled for 10 min was centrifuged for Western blot detection.

### 2.8 Cell counting kit 8 (CCK-8) assay

Cell viability was tested by CCK-8 (Corning Corporation). In short, the PC9 and A549 cells (1 × 10^4^ cells/well) were seeded into 96-well plates. The optical density value at 450 nm was determined 2 h subsequent to the addition of CCK-8 reagent with the utilization of a microplate reader (BioTek).

### 2.9 5-Ethynyl-2′-deoxyuridine (EdU) staining

Cell proliferative capacity was tested with the aid of a Cell-Light EdU DNA Cell Proliferation Kit (RiboBio). After digestion, cells of each group were inoculated into 48-well plates, with 15,000 cells inoculated in each well, and continued to be cultured until the cells were attached to the wall. EdU working solution was added for 48 h culture. Cell nuclei was stained with 4,6-diamidino-2-phenylindole, and after washing with phosphate buffer saline, EdU staining was observed under the inverted fluorescence microscope (Leica). EdU positive cells shows red fluorescence.

EdU positive rate (%) = Number of red fluorescent cells/Number of blue fluorescent cells × 100%.

### 2.10 Glycolysis index detection

Forty-eight hours after transfection, the glucose uptake and production of lactate of PC9 and A549 cells were examined utilizing the glucose uptake colorimetric assay kit and lactic acid colorimetric assay kit (Biovision). Cell oxygen consumption rate (OCR) and extracellular acidification rate (ECAR) were measured using Seahorse XFe96 analyzer (Seahorse Bioscience) using Seahorse XF Glycolysis Stress Test Kit and Seahorse XF Cell Mito Stress Test Kit, respectively.

### 2.11 Statistical analysis

SPSS 22.0 software was used for statistical analysis. All experiments were repeated three times, and the results were expressed as mean value ± standard deviation. The t-test was used to test the difference between the two groups of data, and the one-way analysis of variance was used to test the difference between the multiple groups of data. *p* < 0.05 was statistically significant.

## 3 Results

### 3.1 IGF1R is significantly highly expressed in tissues and cells of LC

**Figure 1:**
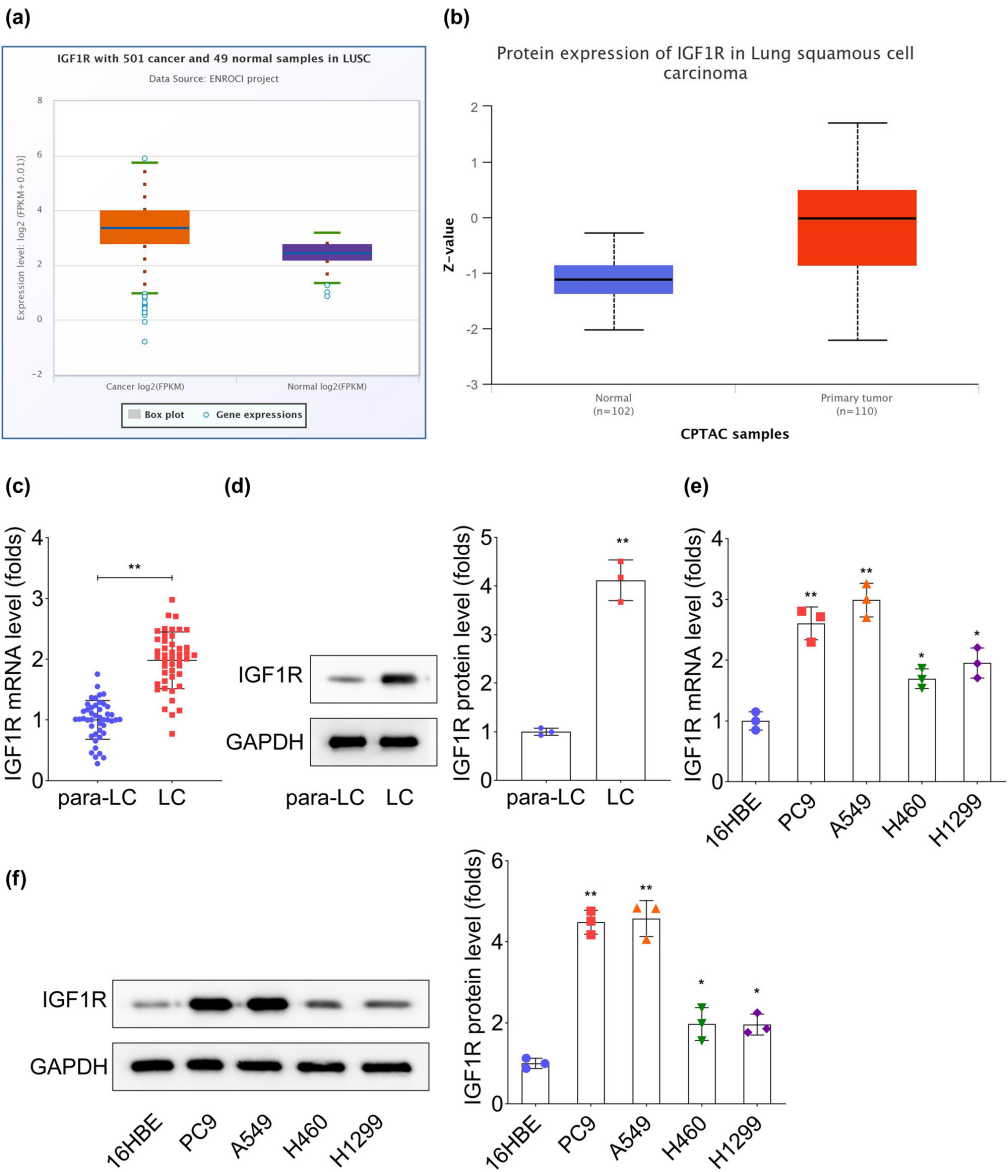
IGF1R is highly expressed in lung cancer (LC) tissues and cells. (a) IGF1R expression in samples of patients with LUSC accessed from the ENCORI. (a) and (b) High protein level of IGF1R in samples of patients with LUSC accessed from the UALCAN Portal. (c) and (d) IGF1R expression levels in LC and para-cancerous tissues were evaluated by RT-qPCR and western blot methods, *n* = 3. (e) and (f) IGF1R expression levels in diverse LC cell lines were evaluated by RT-qPCR and western blot methods, *n* = 3. ^*^ *p* < 0.05, ^**^ *p* < 0.01.

The mRNA expression of IGF1R was analyzed in 501 LUSC tissues and 49 normal tissues from the ENCORI database. The results indicated that IGF1R was significantly overexpressed in LUSC (Figure 1a). Meanwhile, data collected from UALCAN database also verified the high protein level of IGF1R in LUSC (Figure 1b). Subsequently, the outcome was confirmed in clinical specimens, which indicated that IGF1R mRNA (Figure 1c, *p* < 0.01) and protein levels (Figure 1d, *p* < 0.01) were elevated in tissues of LC patients. Meanwhile, both the mRNA and protein levels of IGF1R also upregulated multiple LC cell lines (Figure 1e and f, *p* < 0.05). Furthermore, the upregulation of IGF1R was more pronounced in PC9 and A549 cells, which were chosen for further experiments.

### 3.2 Exogenous lactic acid promotes IGF1R protein stability by enhancing lactylation in LC cells

**Figure 2:**
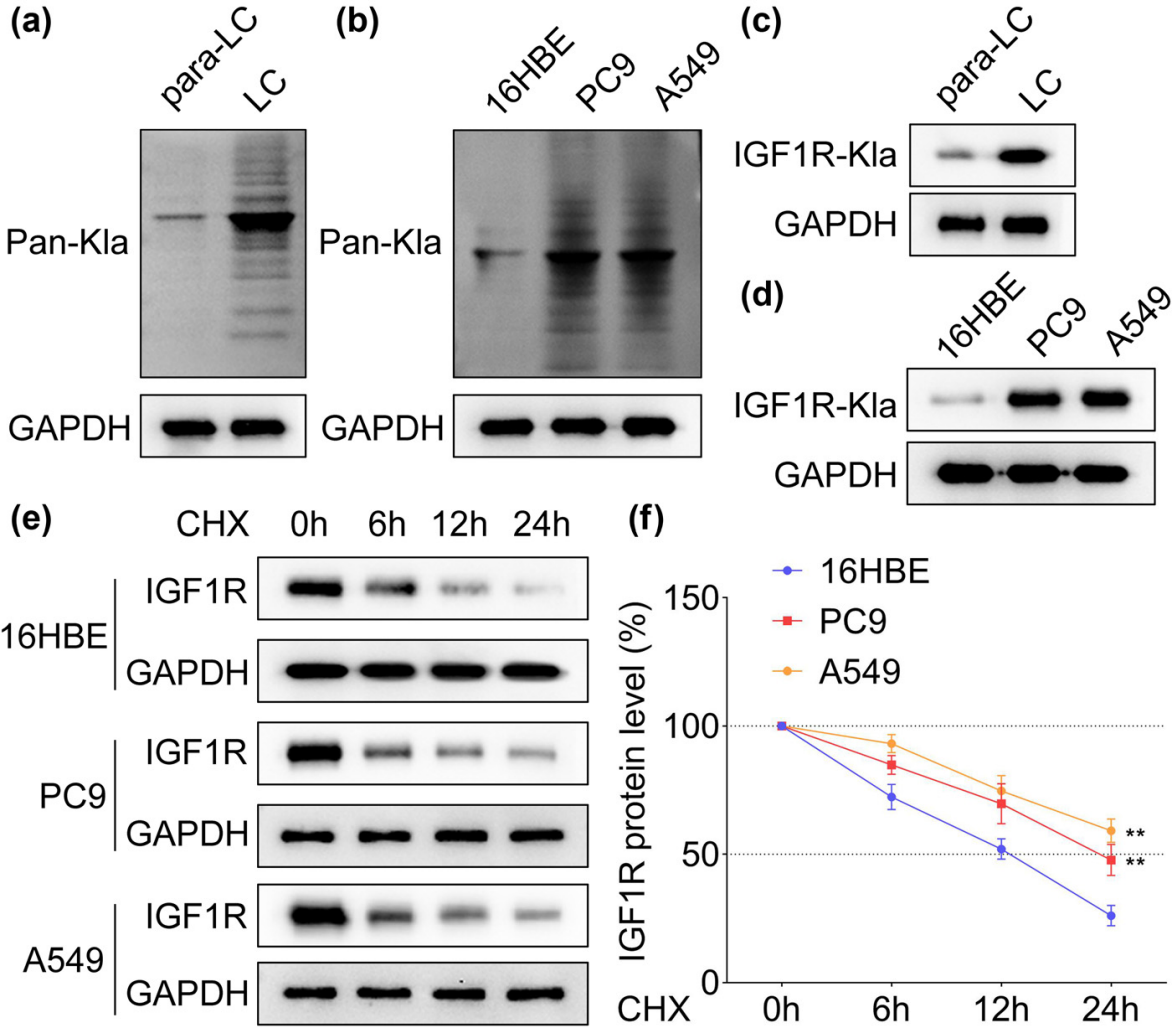
Lactylation regulated the stability of IGF1R protein. The pan lysine lactylation (Pan-Kla) levels of (a) LC and para-cancerous tissues and (b) 16HBE, PC9, and A549 cells were detected by western blot, *n* = 3. IGF1R and lactated IGF1R levels in (c) LC and para-cancerous tissues and (d) 16HBE, PC9, and A549 cells were detected by western blot, *n* = 3. (e) Protein degradation of IGF1R protein in 16HBE, PC9, and A549 cells treated by cycloheximide (CHX, 150 µg/mL) interference, *n* = 3. (f) The quantitative analysis of protein degradation, *n* = 3. ^**^ *p* < 0.01.

**Figure 3:**
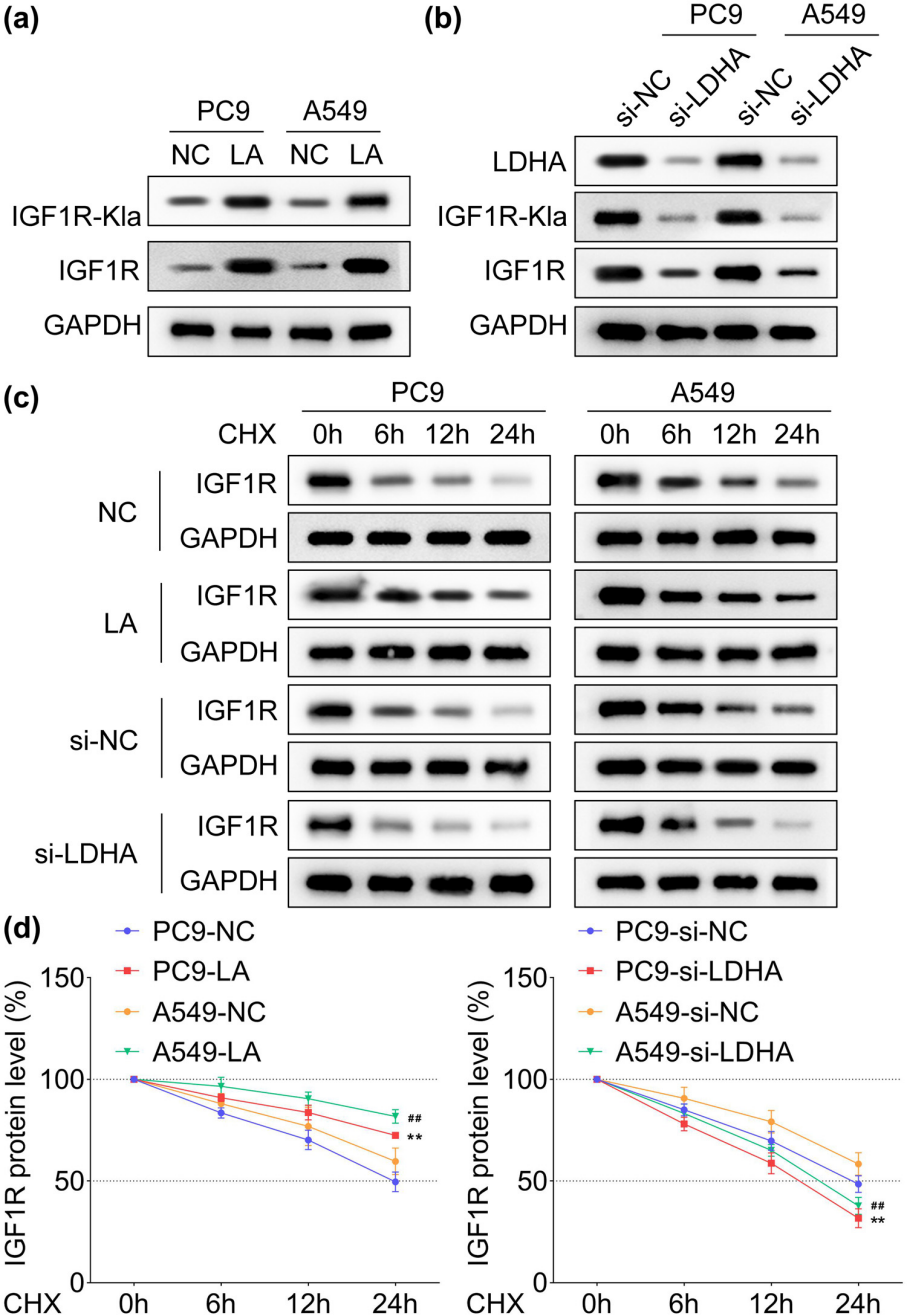
Exogenous lactic acid promotes IGF1R protein stability by enhancing lactylation in LC cells. The protein levels of IGF1R and lactylated IGF1R in PC9 and A549 cells under the interference of (a) the exogenous lactic acid (15 mM) and (b) knockdown of LDHA, *n* = 3. (c) Protein degradation of IGF1R protein in PC9 and A549 cells treated by interference of the exogenous lactic acid (15 mM) and knockdown of LDHA, *n* = 3. (d) The quantitative analysis of protein degradation, *n* = 3. ^**^ *p* < 0.01 (vs PC9-NC and PC9-si-NC), ^##^ *p* < 0.01 (vs A549-NC and A549-si-NC).

**Figure 4:**
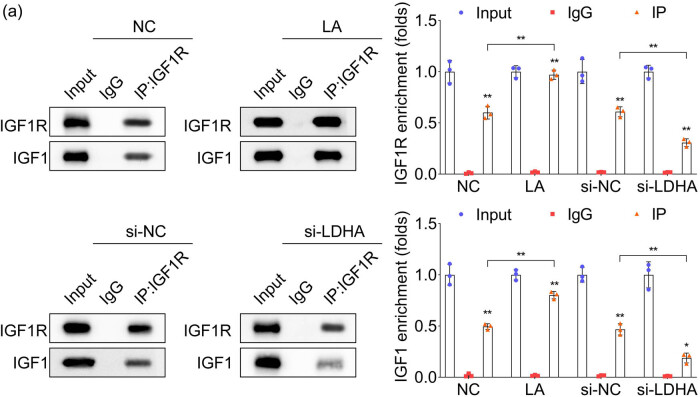
LA enhanced IGF1R and IGF binding. (a) The binding relationship between IGF1R and IGF1 was accessed by Co-IP verified in HEK293T cells, *n* = 3.

Results of pan lysine lactylation (Pan-Kla) levels in LC revealed that the Pan-Kla protein levels were significantly higher in both tissues and LC cell lines (Figure 2a and b), in addition to upregulated lactated IGF1R in LC (Figure 2c and d). The IGF1R protein was found to degrade significantly in PC9 and A549 cells compared with the 16HBE group (Figure 2e and f, *p* < 0.01). It proceeded by utilizing exogenous lactic acid interference and LDHA knockdown performance to promote lactylation in LC cells, respectively, and results showed that the exogenous lactic acid treatment significantly increased the IGF1R protein levels as well as lactated IGF1R in both PC9 and A549 cells, which were significantly decreased following inhibition of LDHA performance (Figure 3a and b). It is pertinent to mention that the IGF1R protein degradation was significantly delayed by exogenous lactic acid application while accelerated by LDHA silencing in both PC9 and A549 cells (Figure 3c and d). Moreover, the tightness of IGF1R binding to IGF1 verified in HEK293T cells was also enhanced by exogenous lactic acid treatment but suppressed by LDHA silencing (Figure 4a).

### 3.3 Lactylation of IGF1R promotes glycolysis in LC cells

**Figure 5:**
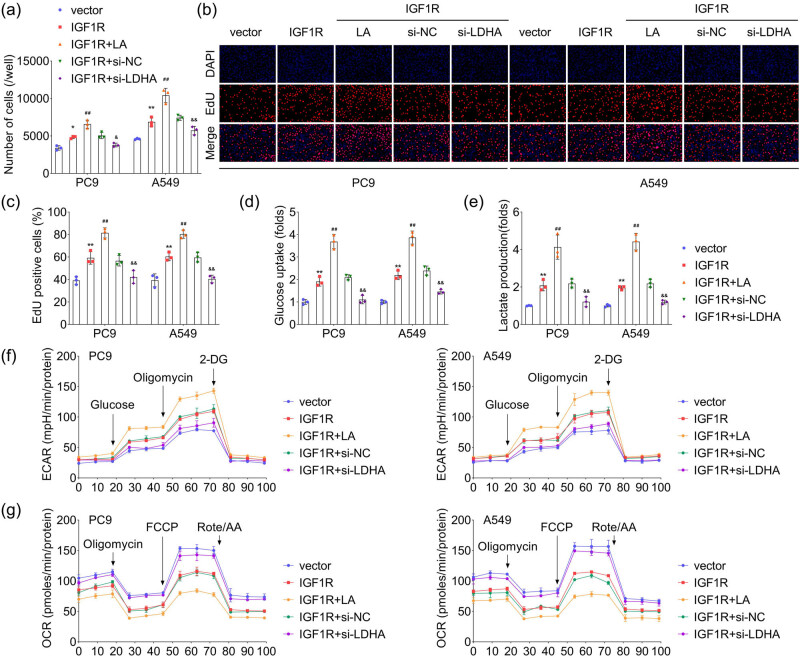
Lactylation of IGF1R promotes glycolysis in LC cells. Under the interference of over-expression of IGF1R, exogenous lactic acid (15 mM) treatment, and LDHA knockdown, (a) the cell viability was accessed by CCK-8 assay, (b) and (c) the cell proliferation was evaluated by EdU staining. Glucose uptake (d) and lactate production (e) in different cells, *n* = 3. (f) and (g) The effects of IGF1R lactylation on extracellular acidification rate (ECAR) and OCR, as determined by glycolysis stress test in PC9 and A549 cells, *n* = 3. ^**^ *p* < 0.01 (vs vector), ^##^ *p* < 0.01 (vs IGF1R), ^&&^ *p* < 0.01 (vs IGF1R + si-NC).

The regulatory effects of IGF1R and its lactylation on cellular functions were further investigated using PC9, A549, and H460 cell lines. Results indicated that overexpressing IGF1R promoted cell viability and proliferation, while exogenous lactic acid treatment translated into further enhancement of the IGF1R gene effects, and knocking down LDHA reduced the IGF1R-induced malignant behaviors in PC9, A549, and H460 cells (Figure 5a–c, Figure S1a–c). Subsequently, the relationship between IGF1R and glycolysis was investigated, and results showed a higher glucose uptake (Figure 5d, Figure S1d) and extracellular lactate (Figure 5e, Figure S1e) in PC9, A549, and H460 cells as a function of IGF1R over-expression and lactic acid treatment, demonstrating the acceleration of IGF1R lactylation on glycolytic metabolism. Moreover, the seahorse assay results exhibited that over-expression of IGF1R and lactic acid treatment increased the ECAR (Figure 5f, Figure S1f) but decreased the OCR (Figure 5g, Figure S1g), while the effects of IGF1R over-expression on glycolysis index were significantly reversed by LDHA knockdown.

## 4 Discussion

**Figure 6:**
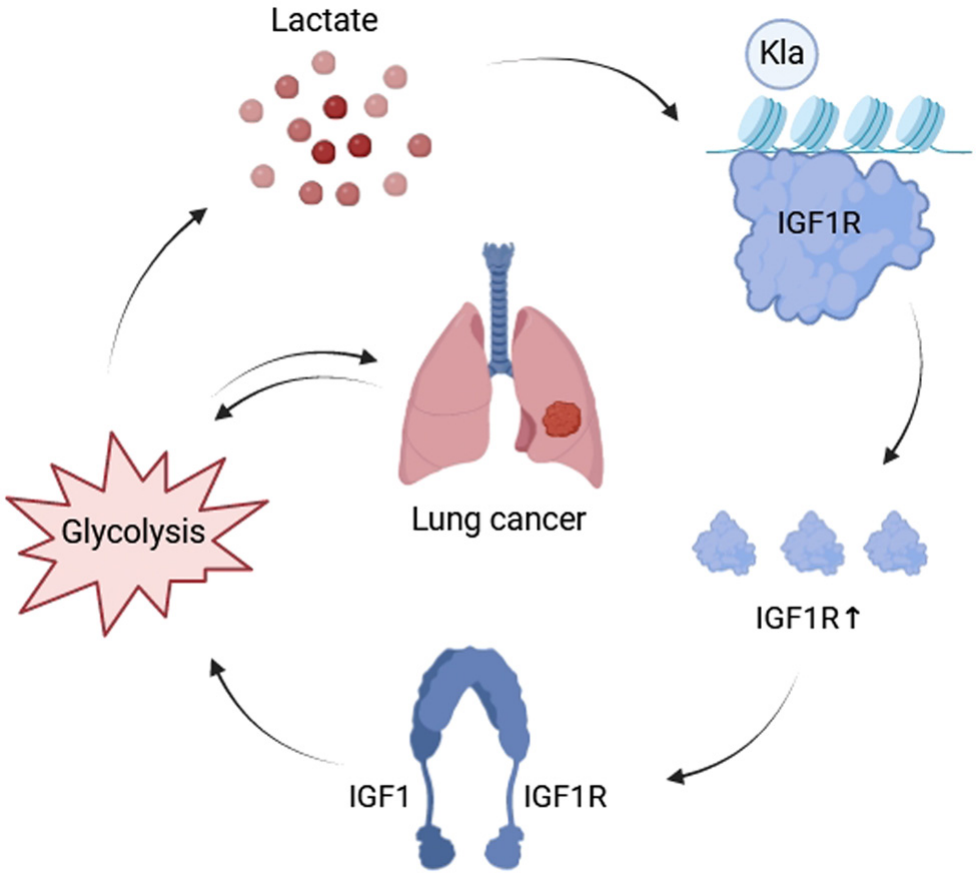
Graphical abstract.

This study revealed that IGF1R was abnormally highly expressed in LC clinical samples and LC cell lines, along with elevated Pan-Kla levels in LC cells, suggesting lactation of IGF1R. Moreover, exogenous lactic acid enhanced lactated IGF1R, improved protein stability, and reversed the effects of LDHA knockdown on glycolysis (Figure 6).

IGF1R is a transmembrane tyrosine receptor protein that promotes cell division by binding to its ligand IGF1, leading to various regulatory effects on tumor cells, such as proliferation, apoptosis, angiogenesis, invasion, and metastasis [15–17]. IGF1R has been found to be abnormally highly expressed in various tumors and was reported to be closely associated with tumor malignant behaviors [6,18]. Our results demonstrated that both the mRNA and protein levels of IGF1R were significantly upregulated in LC tissues and cell lines. IGF1R is known to play a role in regulating glycolysis [19, 20], and it was reported that glycolysis could be one of the malignant phenotypes of LC cells [21]. Additionally, lactic acid, a byproduct of glycolysis, can influence cell metabolism by regulating gene expression through protein lactylation-mediated LC [8]; hence, it was hypothesized that IGF1R was lactated, which was later verified in subsequent experiments.

Lactylation is a recently identified post-translational protein modification that covalently couples lactate groups to protein lysine residues to aid in gene regulation. Mass spectrometry analysis revealed 451 lysine lactation sites on 724 proteins in the human lung, with 141 of these proteins being lactated [22]. It has been reported that lactic acid modulates glycolysis through lactylation-mediated gene expression of glycolytic enzyme HK-1 in LC; the mRNA level of HK-1 was downregulated by lactic acid, and increased by acylation [8]. Our data also found higher Pan-Kla levels in LC tissues and cells, which could probably be induced by IGF1R lactylation. Moreover, the IGF1R protein stability was enhanced in LC cells; it was hypothesized that the stability of IGF1R protein was regulated by lactic acid due to its ability to provide energy to cancer cells through glycolysis. Our results suggested that exogenous lactic acid dramatically increased the IGF1R protein levels and lactated IGF1R, and more importantly, the degradation of IGF1R protein was significantly delayed by exogenous lactic acid, which was in line with previously reported results [23]. LDHA can catalyze the conversion of pyruvate to lactic acid, while the expressions of LDHA were reported to be significantly upregulated in LC tissues and were associated with poor patient prognosis [8]. LDHA further increases histone lactation and leads to metabolic disorders and tumorigenesis of LC cells [23]. This study’s results demonstrated that LDHA knockdown accelerated the degradation of IGF1R protein, as reported before [24].

The binding of ligands to receptors results in receptor oligomerization, activation of protein tyrosine kinase, intermolecular receptor self-phosphorylation, and phosphorylation of cell substrates, ultimately resulting in gene-activated DNA synthesis and cell proliferation. Thus, promoting the binding of IGF1R and IGF1 contributes to cancer progression, a phenomenon observed in various types of cancer [15,25,26]. Results showed that the tightness of IGF1R binding to IGF1 was also enhanced by exogenous lactic acid but suppressed by LDHA silencing.

Over-expression of IGF1R increased cell viability and proliferation, while exogenous lactic acid enhanced the effects of the IGF1R gene, and LDHA knockdown had the opposite effect. IGF1R regulates cell functions like proliferation, differentiation, survival, metabolism, and migration through the activation of different signaling pathways such as PI3K/AKT/mTOR, Ras/Raf/Mek/Erk, and JAK/STAT [27,28]. One limitation of this study is the lack of investigation into the downstream pathway of IGF1R, which requires additional research. Furthermore, the relationship between IGF1R and glycolysis was investigated, where IGF1R lactylation was found to induce glycolytic metabolism in LC. The exogenous lactic acid enhanced the regulatory effect of IGF1R, while the inhibitory effect of LDHA was reversed. Glycolysis-induced lactic acid enhanced the protein stability of oncogene IGF1R through lactylation and promoted glycolysis to generate more lactic acid, creating a self-sustaining cycle. Therefore, targeting IGF1R offers a novel approach for developing therapeutic agents to combat LC.

## 5 Conclusion

This study concluded that IGF1R was extensively lactylated in LC cells, and exogenous lactic acid significantly promoted IGF1R lactylation to stabilize the IGF1R protein. Lactic acid enhanced the impact of IGF1R on glycolysis during lactation, leading to increased lactic acid production and establishing a self-sustaining cycle. Therefore, targeting IGF1R may be a promising therapeutic strategy against LC.

## Supplementary Material

Supplementary Figure
