# Supplementary material for: Lactate-induced IGF1R protein lactylation promotes proliferation and metabolic reprogramming of lung cancer cells
Source: Open Life Sci. 2024 May 29;19(1):20220874. doi: 10.1515/biol-2022-0874 (PMC11151389; doi:10.1515/biol-2022-0874)
Supplement: Supplementary Figure [file biol-2022-0874-sm.pdf]

Supplementary material

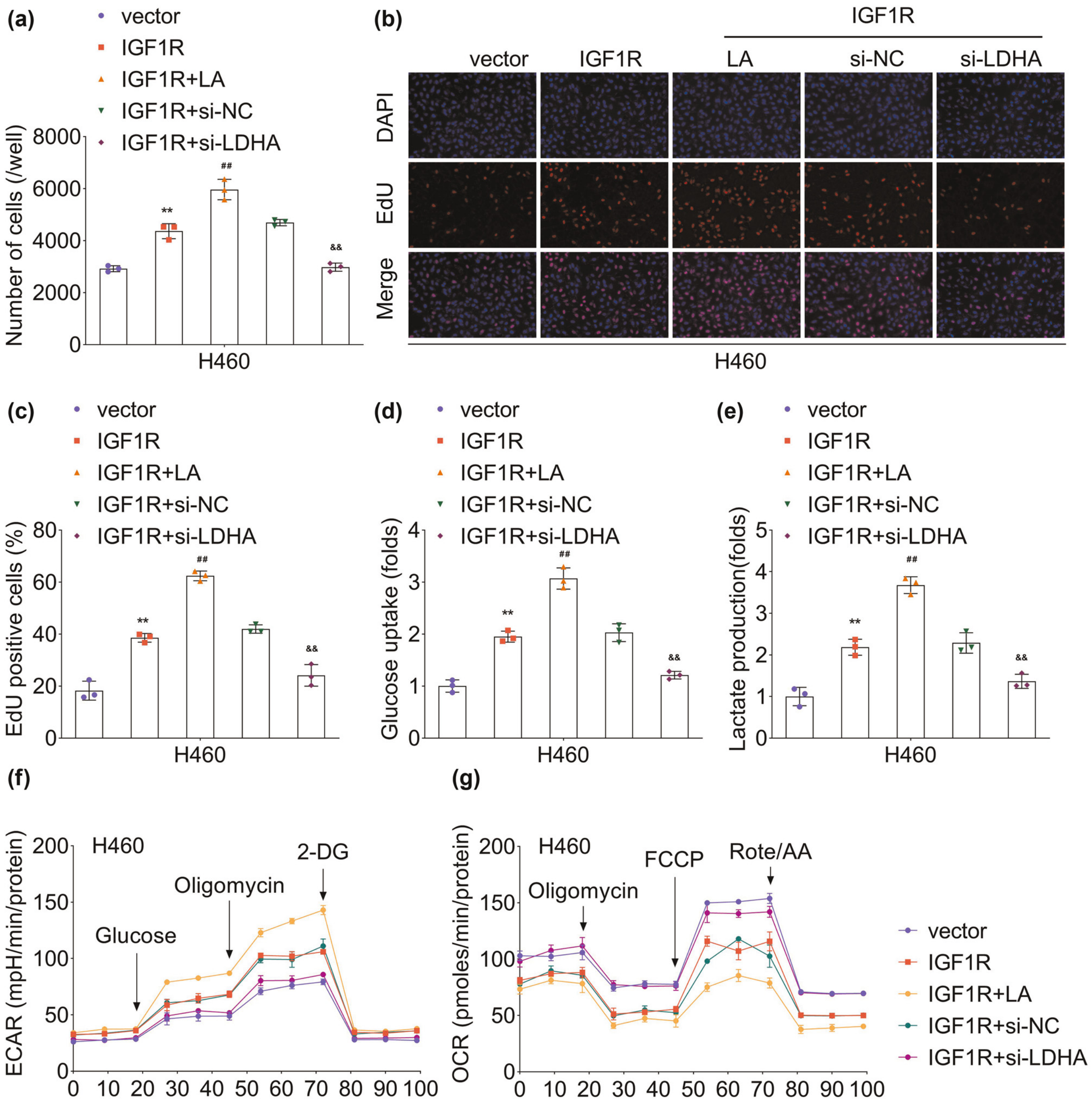

Figure S1: Lactylation of IGF1R promotes glycolysis in LC cells. Under the interference of over-expression of IGF1R, exogenous lactic acid (15 mM) treatment, and LDHA knockdown, (a) the cell viability was accessed by CCK-8 assay, (b) and (c) the cell proliferation was evaluated by EdU staining. Glucose uptake (d) and lactate production (e) in different cells, $n = 3$. (f) and (g) The effects of IGF1R lactylation on extracellular acidification rate (ECAR) and OCR, as determined by glycolysis stress test in PC9 and A549 cells, $n = 3$. $^{**}p < 0.01$ (vs vector), $^{\#\#}p < 0.01$ (vs IGF1R), $^{\&\&}p < 0.01$ (vs IGF1R + si-NC).
